# Izydor Fajersztajn-Krzemicki (1867–1935)

**DOI:** 10.1007/s00415-019-09606-4

**Published:** 2019-10-31

**Authors:** Sławomir Gonkowski, Jaroslaw Calka

**Affiliations:** Departement of Clinical Physiology, Faculty of Veterinary Medicine, University of Warmia and Mazury in Olsztyn, ul Oczapowskiego 13, 10-718 Olsztyn, Poland

**Fig. 1:**
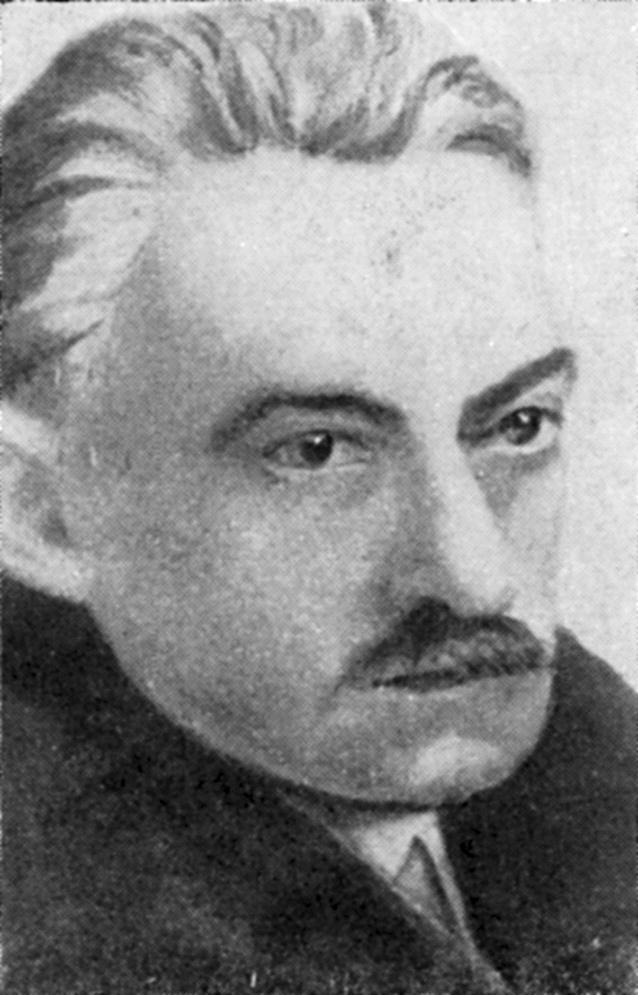
Izydor Fajersztajn-Krzemicki. Photo from public domain

Izydor Fajersztajn-Krzemicki (Fig. 1) was born in a Jewish family on September 25, 1867, in Warsaw, which at that time was a part of the Russian Empire [1]. In 1886, he passed the secondary-school-leaving exam in his hometown [2] and started to study medicine at Warsaw University, receiving his medical degree in 1891 [1]. Soon afterwards he went to Cracow, which at that time was located within the borders of the Austro-Hungarian Empire. In Cracow, Fajersztajn-Krzemicki continued his studies at the Jagiellonian University, where in 1893 he received a doctoral diploma in medical sciences [1, 3, 4].

In the same year, Izydor Fajersztajn-Krzemicki travelled around Europe and deepened his knowledge of medicine. He worked at the clinics directed by Richard von Krafft-Ebing in Vienna and Adolf von Strümpell in Erlangen [1]. In 1894, he went to Lvov, where he lived and worked for the rest of his life. Initially, he worked as an assistant surgeon at the Public Hospital [3].

In 1898, Fajersztajn-Krzemicki became the head of the Department of Nervous Illnesses at the Public Policlinic, and soon afterwards the director of all policlinics [4]. He held this function until 1934, when he resigned for health reasons. Moreover, from 1918 to his death, he was head of a sanatorium for patients with mental and neurological diseases [1, 3]. Izydor Fajersztajn-Krzemicki died on February 3 or 4, 1935, in Lvov [1, 5].

Fajersztajn-Krzemicki was not only a neurology practitioner, consummate clinician and diagnostician, but also an inquisitive scientist. The first experiments he conducted during his studies at Warsaw University [1], under the direction of the famous histologist Henryk Hoyer, studied innervation of the frog and obtained results published in 1889 in the Journal of Warsaw Medical Association [1].

However, Fajersztajn-Krzemicki has gone down in the history of neurology thanks to his later studies. He was a pioneer of the histopathology of the nervous system. In 1901, he was the first to describe the silver staining method of visualization of nerve fibers in an article in German entitled “Ein neues Silberimprägnationsverfahren als Mittel zur Färbung der Axencylinder” (A new silver impregnation process as a means of coloring the axons), which was published in the journal “Neurologisches Zentralblatt” [6]. Soon afterwards this method was slightly modified by the German neurologist Max Bielschowsky (1869–1940) [7] and the use of silver staining to visualise nerves is universally known as Bielschowsky stain [1].

Fajersztajn-Krzemicki was a modest person and he did not fight for the name of discoverer of this method. As mentioned by another Polish neurologist, Kazimierz Orzechowski, Fajersztajn-Krzemicki was content with the letter from the distinguished neuroscientist Ludwig Edinger (1855–1918) [8], written just after the publication of Bielchowsky’s modification [4], in which Edinger claimed that Fajersztajn-Krzemicki was the discoverer of this method [1, 4].

Fajersztajn-Krzemicki was one of the first scientists to use Röntgen radiation to photograph the structures located in the brain, as well as constructing (according to his invention) a macrotome to do cross sections of this organ [1, 3]. However, the surname of Fajersztajn-Krzemicki went into the pages of neurology textbooks due to his studies on reflexes during spinal cord injury. Namely, in 1901, he described for the first time the crossed symptom, also known as “well leg-raising test,” during sciatica [1]. This symptom is observed in unilateral sciatica when raising the healthy (“well”) leg causes the pain in the symptomatic (“sick”) leg, which is not raised. The pain is caused because raising the healthy leg causes tension in the nerve root not only on this side of the body but also along the midline of the cauda equina and in the contralateral nerve roots [9]. Fajerszatajn-Krzemicki described this observation in the article “Ueber das gekreuzte Ischiasphänomen. Ein Beitrag zur Symptomatologie der Ischias (About the crossed sciatica phenomenon. A contribution to the symptomatology of sciatica) published in journal “Wiener klinische Wochenschrift” [10]. At the present time, this symptom is sometimes called the “Fajersztajn test,” “Fajersztajn-Krzemicki test,” or the “well leg raising test of Fajersztajn” and is considered to be an upgraded form of Lasègue's sign [1, 3, 9].

In the 1930s, Izydor Fajersztajn-Krzemicki practised ophthalmology. He examined visual fields using an instrument called a “campimeter” and developed his own formula to study the blind spot [1]. Moreover, Fajersztajn-Krzemicki constructed an apparatus which he called an “equatorial pregometer,” enabling the detection of even minor disorders in the extraocular muscles [1].

Due to the achievements of Fajersztajn-Krzemicki, after his death, the Polish neurologist Jakub Rothfeld wrote “Polish neurology lost one of the bravest worker. Krzemicki was a comprehensive mind with a wide range of interests in the area of medicine, physics, biology and mathematics. As a scientist he was precise, critical and demanding, especially with himself” [3].
